# Lung lipids associated with smoking and ECIG use in a cross-sectional study and clinical trial

**DOI:** 10.1186/s12931-025-03267-w

**Published:** 2025-05-20

**Authors:** Joseph P. McElroy, Min-Ae Song, John R. Barr, Michael S. Gardner, Garret Kinnebrew, Zsuzsanna Kuklenyik, Jennifer D. Kusovschi, Jon C. Rees, Benjamin C. Blount, MuChun Tsai, Mark D Wewers, Sahar Kamel, Sarah A. Reisinger, Amarnath Singh, Daniel Y. Weng, Peter G. Shields

**Affiliations:** 1 Center for Biostatistics, Department of Biomedical Informatics, College of Medicine, The Ohio State University, Columbus, OH USA; 2 Division of Environmental Health Sciences, College of Public Health, The Ohio State University, Columbus, OH USA; 3 Comprehensive Cancer Center, The Ohio State University and James Cancer Hospital, 460 W. 10 th Avenue, 9 th Floor, Suite D920, Columbus, OH 43210-1240 USA; 4 Division of Laboratory Sciences, Centers for Disease Control and Prevention, National Center for Environmental Health, Atlanta, GA USA; 5 Department of Biomedical Informatics, College of Medicine, The Ohio State University, Columbus, OH USA

**Keywords:** Smoking, Electronic Cigarette, Lipidomics

## Abstract

**Background:**

While electronic cigarettes (ECIG) may have lower toxicant delivery than cigarettes, ECIG-liquids and aerosols still contain toxicants that can potentially disrupt lung lipid homeostasis.

**Methods:**

Participants from two studies underwent bronchoscopy and bronchoalveolar lavage (BAL). Ninety-eight participants (21-44 years old) were included in a cross-sectional study, with 17 ECIG users, 52 non-smokers, and 29 smokers. In the four-week clinical trial, 30 non-smokers were randomly assigned to use nicotine-free, flavorless ECIG or no use. A panel of 75 quantifiable lipid species and 7 lipid classes were assessed in the BAL using two tandem mass spectrometry (MS/MS) platforms. Ten cytokines and lipid-laden macrophages (LLM) were analyzed using the V-PLEX Plus Proinflam Combo 10 panel and Oil Red O staining, respectively.

**Results:**

In the cross-sectional study, 43 lipids were associated with smoking status at FDR<0.1, including two between ECIG and non-smokers (PC(14:0/18:1) and PC(18:0/14:0)) in pairwise follow-up analyses (Bonferroni-adjusted p<0.017). Associations between lipid species and cotinine, inflammatory markers, including IL-1β and IL-8, and LLM were also identified, as well as differences in lipid classes between smokers and the other groups. Smokers had higher saturated lipids, including ceramide (CER), sphingomyelin (SM), and diacylglycerol (DAG) than that of non-smokers and ECIG users. No significant associations were identified in the 4-week clinical trial.

**Conclusions:**

Smoking was associated with altered lipid levels, as compared to both non-smokers and ECIG users; the majority were downregulated and ECIG effects tend to be smaller in magnitude than smoking effects, although some were different than those in the smokers group. This is a novel study of healthy individuals examining lipidomic differences between smokers, ECIG users, and non-smokers, indicating potential roles of smoking and ECIG-related lipid alterations in pulmonary disease.

**Trial registration:**

The study was approved by The OSU Institutional Review Board (OSU-2015C0088) in accordance with its ethical standards, the Helsinki declaration, and the Belmont Report, and is registered on Clinicaltrials.gov (NCT02596685; 2015-11-04).

**Supplementary Information:**

The online version contains supplementary material available at 10.1186/s12931-025-03267-w.

## Introduction

Cigarette smoke contains over 6,000 constituents, many of which cause respiratory disease, cardiovascular disease, and cancer, and is the leading cause of preventable death [1]. Electronic cigarette (ECIG) use could potentially represent a risk reduction strategy for smokers due to much lower levels of toxicants and carcinogens [2, 3]. However, ECIGs may have unique toxicities for both smokers and non-smokers. The primary solvent carriers of ECIG liquids are propylene glycol (PG) and vegetable glycerin (VG). While the Food and Drug Administration generally recognizes them as safe for oral use, studies show potential adverse effects in the lungs when heated and aerosolized. Recent studies, including ours, indicate an ECIG toxic effect on the lung, including an altered lung immune response [4–11].

Exposure to ECIG aerosols disrupts normal lung lipid homeostasis in laboratory animals and humans [7, 12]. A study of ECIG-exposed mice found accumulated lipid-laden macrophages (LLM) and phospholipids in bronchoalveolar lavage (BAL) cells [12]. Our previous cross-sectional study of smokers, ECIG users, and non-smokers showed higher levels of LLM in almost all smokers and half of the ECIG users, while none was found in non-smokers [7]. Interestingly, we observed LLM to be significantly associated with cytokines such as IL-4 and IL-10 in ECIG users only [7].

Lipids are essential for lung biology by playing a role in cell membrane structure integrity, signaling transduction, surfactant synthesis, and inflammation [13, 14]. Lipid-derived metabolites mediate cellular inflammation, and altered lipid homeostasis has been implicated in various respiratory diseases, including asthma, chronic obstructive pulmonary disease (COPD), and lung cancer [14–18]. Evidence from targeted blood lipids analyses shows cigarette smoking-associated lipid alterations [19–22]. For example, smokers from the National Health and Nutrition Examination Survey had lower serum high-density lipoprotein (HDL) and higher serum triglycerides compared to non-smokers [19]. Similarly, recent efforts have been made to associate ECIG with lipids in blood samples, but with conflicting findings [20, 23, 24]. Targeted approaches found lower HDL and higher triglycerides [20, 24] in ECIG users compared to non-smokers, while an untargeted lipidomic analysis did not find any ECIG-associated lipids but distinct sex-specific alterations in lipid species [23].

With the limited studies cited above, the effects of ECIG on lung lipids remain unclear. Notably, there is a wide diversity of lipid abundance and species across tissues [25]. Thus, it is essential to evaluate comprehensive lipidome profiles in the target organ, such as the lung in order to provide insights into the direct effects of cigarette smoking and ECIG use on lipids. Considering the potentially important role of lipids in respiratory diseases and the lack of understanding of target organ effects on lipid metabolism for cigarette smoking and ECIG, we examined the lung lipidome of smokers, ECIG users, and non-smokers in a cross-sectional study and a clinical trial of non-smokers trained to vape nicotine-free, flavorless ECIGs. Also, we examined how these lipids are associated with smoking and EC use intensities to examine dose-dependent effects using urinary biomarkers of exposure and further related to LLM and inflammatory responses to understand potential biological implications of smoking/ECIG use-related lipids. We hypothesize that smoking and ECIG use disrupt the lung lipidome, impacting immune responses and potentially heightening the risk for pulmonary immune responses.

## Methods

### Participants and design

#### Cross sectional study

Participants (n=98; 17 ECIG users, 52 non-smokers, 29 smokers; age range: 21–44 years old) underwent bronchoscopy and bronchoalveolar lavage (BAL). They were recruited from 2015-2019 through multiple outlets, including Craigslist, media (print, television, and radio), The Ohio State University (OSU) Study Search website, and the participant registry for the OSU Tobacco Centers of Regulatory Science. Additional human subject details have been previously published. [26]

The smoking group was comprised of exclusive and active cigarette smokers with a current use of 10 or more cigarettes/day for at least 6 months. Non-smokers were defined as those who had smoked less than 100 cigarettes over their lifetime (the CDC definition [27]) and did not have an ECIG for a year or more prior to the study. ECIG users were those who used ECIGs daily for at least a year with no cigarette smoking for at least 5 months. Fourteen out of 17 of the ECIG users were former smokers. Smoking status was verified using the NicAlert^®^ cotinine test strip (Nymox Pharmaceutical Corporation, St. Laurent, QC, Canada) with a test level threshold of one.

Medical exclusion criteria are listed in Document S1. Additional exclusion criteria were pregnancy, self-reported marijuana or other combustible tobacco use within three months of bronchoscopy, self-reported >10 times smoking marijuana in their life, bronchoscopy or other lung procedure within 6 months, known allergies to study medications, general anesthesia in the past year, or used inhalant medications. Cannabis use was assessed biochemically. Informed consent was obtained from all participants prior to participation. The study was approved by The OSU Institutional Review Board (OSU-2015 C0088) in accordance with its ethical standards, the Helsinki declaration, and the Belmont Report, and is registered on Clinicaltrials.gov (NCT02596685; 2015-11-04). The analysis of deidentified samples by the CDC was not deemed involvement in human subject research.

Bronchoscopies were scheduled within two weeks following an orientation session, randomized to either the right or left lung. Subjects were lightly sedated (intravenous) as needed for the procedure. BAL samples were recovered by wedging the bronchoscope into a subsegmental bronchus and flushing with saline (20 ml) approximately 5–7 times.

Study data were collected and managed using REDCap electronic data capture tools hosted at OSU [28, 29]. REDCap (Research Electronic Data Capture) is a secure, web-based software platform designed to support data capture for research studies, providing 1) an intuitive interface for validated data capture; 2) audit trails for tracking data manipulation and export procedures; 3) automated export procedures for seamless data downloads to common statistical packages; and 4) procedures for data integration and interoperability with external sources.

#### Clinical trial

Thirty non-smokers from the cross-sectional study were randomized to nicotine-free, flavorless ECIG use or no use controls for ethical reasons and for investigating the biological effects of the primary constituents of ECIG (i.e., PG and VG). Additional human subject details have been previously published [30]. One week post baseline bronchoscopy, the ECIG randomized participants were provided with nicotine-free, flavorless Innokin iTaste VV 4.0 refillable tank ECIG device (https://www.innokin.com/) and e-liquids that contained 50% PG and 50% VG (http://www.ecblendflavors.com/flavorless-eliquid/) [8]. Subjects were trained in using the device, instructed to use the device for 20 puffs over 60 minutes at least twice per day, and send a photo of the LED display screen with puff counts daily. The second bronchoscopy was performed five weeks after the baseline bronchoscopy, which was four weeks of ECIG use for the treated group. Use was confirmed via an increase in urinary PG. Power for the clinical trial was calculated prior to the beginning of the study: Considering a significance threshold corresponding to one expected false discovery per 1000 tests (p=0.001), a two-sided, paired t-test achieves 80% power to detect a mean of paired differences equal to 0.8 standard deviations of those paired differences.

### Cytokines

Cytokines were measured in the BAL with the V-PLEX Plus Proinflam Combo 10 panel (Meso Scale Discovery, Rockville, MD) following manufacturer’s instructions, which measured IFN-G, IL-1B, IL-2, IL-4, IL-6, IL-8, IL-10, IL-12p70, IL-13, and TNF-a. Cytokine features were an average of duplicate measures.

### Lipid-laden macrophages (LLM)

Cells were fixed onto glass slides after centrifuging cytospin slides made from BAL (500 µl). After rinsing with distilled water, fixed slides were stained with Oil Red O (Pfaltz and Bauer, Inc., Waterbury, CT) for 15 minutes, and re-rinsed with distilled water. After hematoxylin staining (Thermo Scientific^TM^ Richard-Allan, Waltham, MA), ammonia water was used to process blue sections. Slides were rinsed with tap water after each step [31, 32].

### Lipids

#### Liquid chromatography tandem mass spectrometry (LC-MS/MS) lipidomics

Validation of LC-MS/MS methods for lipids has been described previously [33, 34]. Both methods employed a one-pot, dry-pellet extraction in a 96-wellplate format. For the quantification of phospholipids (PL), 20 µL of BAL fluid was used. For LC-MS/MS analysis of FC and CE, 50 µL of 100x diluted plasma and 50 µL of AF4-separated plasma fractions were used, or 50 µL of 5x diluted CSF and 200 µL of AF4-separated CSF fractions. One deuterium-labeled analog per lipid class (phosphatidylcholine (PC), lyso-phosphatidylcholine (LPC), phosphatidylethanolamine (PE), phosphatidylinositol (PI), and sphingomyelin (SM)) was used as the internal standard (Avanti Polar Lipids, AL, USA). LC-MS/MS peak area ratio-based calibration and quantification were accomplished using a value-assigned plasma pool diluted into the linear range of the instrument, with calibrator dilution factors ranging from approximately 30 to 3000. The limits of quantification in BAL fluid were 369 ng/mL for PC, 81 ng/mL for SM, 29 ng/mL for PE, and 9 ng/mL for PI. A targeted LC-MS/MS approach with unit resolution was applied to monitor PL species commonly found in human plasma (19 for PC, 19 for PE, 8 for LPC, 15 for PI, and 16 for SM). The LC separation was HILIC mode, where lipids are separated largely by head-group, with fatty-acids having little impact on retention. Thus, lipid class peaks could be generated by summing individual species signals and quantified separately from the individual species. Individual PL species with total FA carbon chain lengths and total double bonds were identified based on species-specific molecular ions and generic head group daughter ions. Note that this approach does not allow experimental verification of fatty acyl chains that belong specifically to the sn1 and sn2 positions. PC(32:0), the most abundant lipid in lung surfactant, was separately reported with a limit of quantification of 1.3 ng/mL.

For LC-MS/MS analysis of nonpolar lipids cholesteryl ester (CE), triacylglycerol (TAG), and free cholesterol (FC), 50 µL of BAL fluid was used. A single deuterated internal standard was employed for each lipid class (CE and TAG), as well as for unesterified (free) cholesterol (FC). Calibrators and QCs were prepared by diluting the human serum pools of NIST SRM-1951c into the linear range of the instrument, with dilution factors ranging from approximately 30 to 3000. The LC separation was in the normal-phase mode, which also separates lipids by class (head-group) rather than fatty-acid species. Atmospheric pressure chemical ionization (APCI) coupled with in-source fragmentation was employed in the mass spectrometer prior to MS/MS, allowing the CE and TAG classes to be quantified as a single signal (as well as FC), however species information for CE and TAG is not available with this approach.

#### Flow-Injection differential mobility scanning tandem mass spectrometry (FIA-DMS-MS/MS) lipidomics

Lipids from BAL samples were extracted using a modified Bligh/Dyer method [35]. 2.0 mL methanol, 1.0 mL dichloromethane (DCM), 0.6 mL water, and 25 µL internal standard mixture (AB Sciex, P\N 5040156) were added to 0.4 mL BAL and vortex mixed. Samples were allowed to stand for 30 min, followed by another addition of 1.0 mL DCM and 1.0 mL water. To enhance phase separation, samples were centrifuged for 10 min at 1200 rpm, followed by removal of the lipid-containing bottom layer. An additional 2.0 mL DCM was added to each sample and a second extraction was performed with the lower lipid-containing portion combined with the first extraction. Samples were evaporated to completion under a stream of nitrogen and reconstituted in 0.3 mL of 50:50 (v:v) DCM:methanol containing 10 mM ammonium acetate.

A targeted lipidomics analysis was conducted using the Lipidyzer platform. A 50 µL aliquot was infused into the mass spectrometer twice using two different methods. The first method uses a SelexION Differential Mobility Spectrometry (DMS) analyzer with 1-propanol as a gas modifier to separate and analyze species in the phosphatidylcholine (PC), phosphatidylethanolamine (PE), sphingomyelin (SM), lyso-phosphatidylcholine (LPC), and lyso-phosphatidylethanolamine (LPE) lipid classes. A second method disables the DMS and analyzes lipid species in the cholesteryl ester (CE), ceramide (CER), diacylglycerol (DAG), free fatty acid (FFA), and triacylglycerol (TAG) classes. A full list of lipid species analyzed along with MRM transitions can be found in Su et. al (2021) [36]. A quality control serum sample was included with each batch to ensure instrument reliability and establish method reproducibility. Data processing was conducted using the Lipidyzer Lipidomics Workflow Manager software, including lipid species identification and concentration calculation in nmol/mL. Calculations are based on isotope dilution principles, multiplying mean signal area ratios with the nmol amount of corresponding isotope-labeled internal standard in the infused extracts and dividing by the volume of BAL extracted.

### Statistical methods

All statistical analyses were performed in R [37] unless otherwise noted. For demographic variables (Table 1), Fisher’s exact test was employed for categorical variables and Kruskal-Wallis test for continuous variables. In analyses with lipidomics features as the dependent variable, left censored log-normal models [38] were employed because of limit of detection values (LOD = 0.011875) and right skewing of the data using the ‘survreg’ function from the survival package [39]. Linear models were employed for other continuous dependent variables. For main effects with more than two levels (smoking status), ANOVA was performed on the models to test the overall effect (omnibus model), followed by pairwise t-tests. Because of the presence of batch effects, for the cross-sectional analyses of LLM, the LLM variable considered was the residuals from a linear model with LLM as the dependent variable and batch as the independent variable.

**Table 1:** Demographic variable distribution across smoking groups. ECIG = E-cigarette

	**level**	**Non-Smoker**	**ECIG user**	**Smoker**	**p**
**n**		52	17	29	
**Race (%)**	**Black**	3 (5.8)	1 (5.9)	1 (3.4)	0.864
	**White**	38 (73.1)	14 (82.4)	24 (82.8)	
	**Other/Missing**	11 (21.2)	2 (11.8)	4 (13.8)	
**Gender (%)**	**Female**	33 (63.5)	6 (35.3)	7 (24.1)	0.002
	**Male**	19 (36.5)	11 (64.7)	22 (75.9)	
THC (%)*	**Neg**	38 (90.5)	12 (75.0)	10 (43.5)	<0.001
	**Pos**	4 (9.5)	4 (25.0)	13 (56.5)	
**BMI median [IQR]**		24.44 [22.35, 27.14]	25.98 [24.25, 27.54]	24.37 [21.03, 27.89]	0.196
**Age median [IQR]**		25.00 [23.00, 27.00]	27.00 [25.00, 29.00]	26.00 [25.00, 29.00]	0.085
**Pack Years median [IQR]**		0.00 [0.00, 0.00]	3.60 [0.15, 6.38]**	9.00 [4.50, 11.70]	<0.001
**Cigarettes/day median [IQR]**		0.00 [0.00, 0.00]	15.00 [1.35, 20.00]**	20.00 [12.00, 20.00]	0.045

Fisher’s Exact Test for categorical variables; Kruskal-Wallis Rank Sum Test for Continuous

^*^ delta-. 9-tetrahydrocannabinol

^**^ All but three ECIG users were former smokers

In the analysis of the clinical trial lipidomics data, follow-up lipidomics measure was the dependent variable, and a covariable baseline measure was included in the models. For models where the main effect was another measure such as propylene glycol (rather than randomized treatment assignment: ECIG vs. control), the main effect in the model was follow-up – baseline for the measure. Because of the presence of batch effects in LLM, in the clinical trial, this variable is the difference in the residuals between follow-up and baseline from models with LLM as the main effect and batch as the independent variable.

False discovery rate (FDR) [40] of 0.1 is considered as the threshold for significance for the primary models. FDR was computed separately for the multivariable models and the omnibus models, as well as for each general hypothesis (e.g., lipid vs. LLM, lipid vs. smoking group, lipid vs. biomarkers of inflammation (IM)). For follow-up pairwise tests, Bonferroni corrected [41] p = 0.017 (0.05/3 tests) is considered for significance. Follow-up multivariable models for significant lipidomics features included gender, THC (delta-. 9-tetrahydrocannabinol) positivity, and age. Because the objective of the multivariable models was to explore the nature of the already identified associations, comparison-wise p=0.05 is considered as the significance threshold. For lipid ontological analyses, the web-based LION [42] software was employed using ‘Target List’ mode with significant features (FDR < 0.05), and the 75 lipidomics features as background. FDR < 0.05 was used to select associated features for the LION analyses because for some comparisons > 50% of lipids were significant at FDR < 0.1. For significance of LION ontological class, FDR < 0.1 was considered as the significance threshold.

## Results

### Demographics (Cross Sectional)

**Fig. 1:**
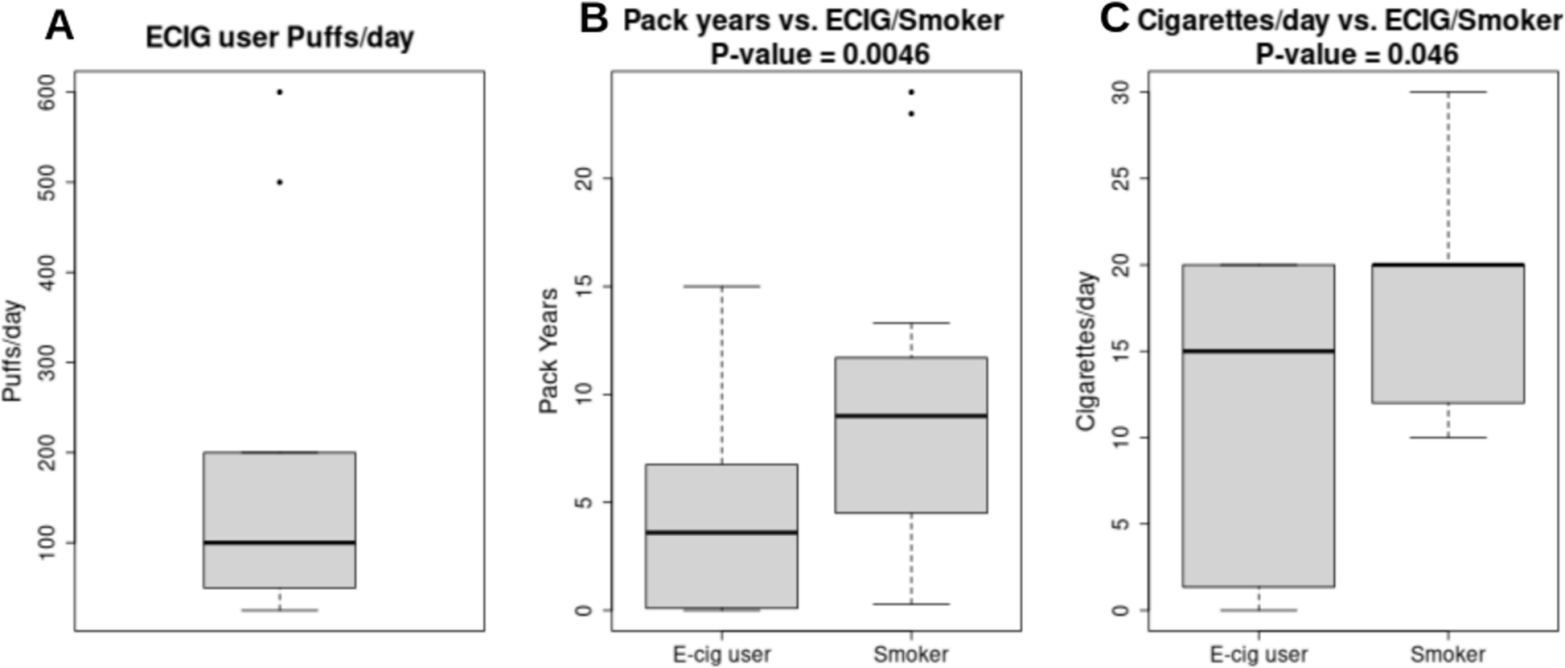
Boxplots of cross-sectional ECIG (E-cigarette) user puffs per day (**A**), pack years (**B**) and cigarettes per day (**C**) in E-cig (E-cigarette users) vs. Smokers. The center line denotes the median, while the box contains the 25^th^ to 75 th percentile values. *P*-values are from the Wilcoxon rank sum test

Table 1 shows the demographic characteristics of the smoking group for the cross-sectional data (17 ECIG users, 52 non-smokers, and 29 smokers). For interpretation of results, it should be noted that non-smokers were instructed to take 40 puffs per day in the clinical trial, whereas the ECIG users in the cross-sectional study averaged about 160 puffs per day (Fig. 1A). Also for interpretation of results, it should be noted that 14 out of 17 of the ECIG users were former smokers. Pack years and cigarettes per day were significantly lower in ECIG users than in cigarette smokers (Wilcoxon rank sum p=0.005 and p=0.046, respectively; Fig. 1B and C, respectively). Gender, urine THC positivity, and age are at least marginally associated (p < 0.1) with the smoking group, and so was included in the multivariable models. Baseline data from the clinical trials’s 30 non-smokers are included in the cross sectional study and in Table 1.

### Lipidomics data

**Fig. 2:**
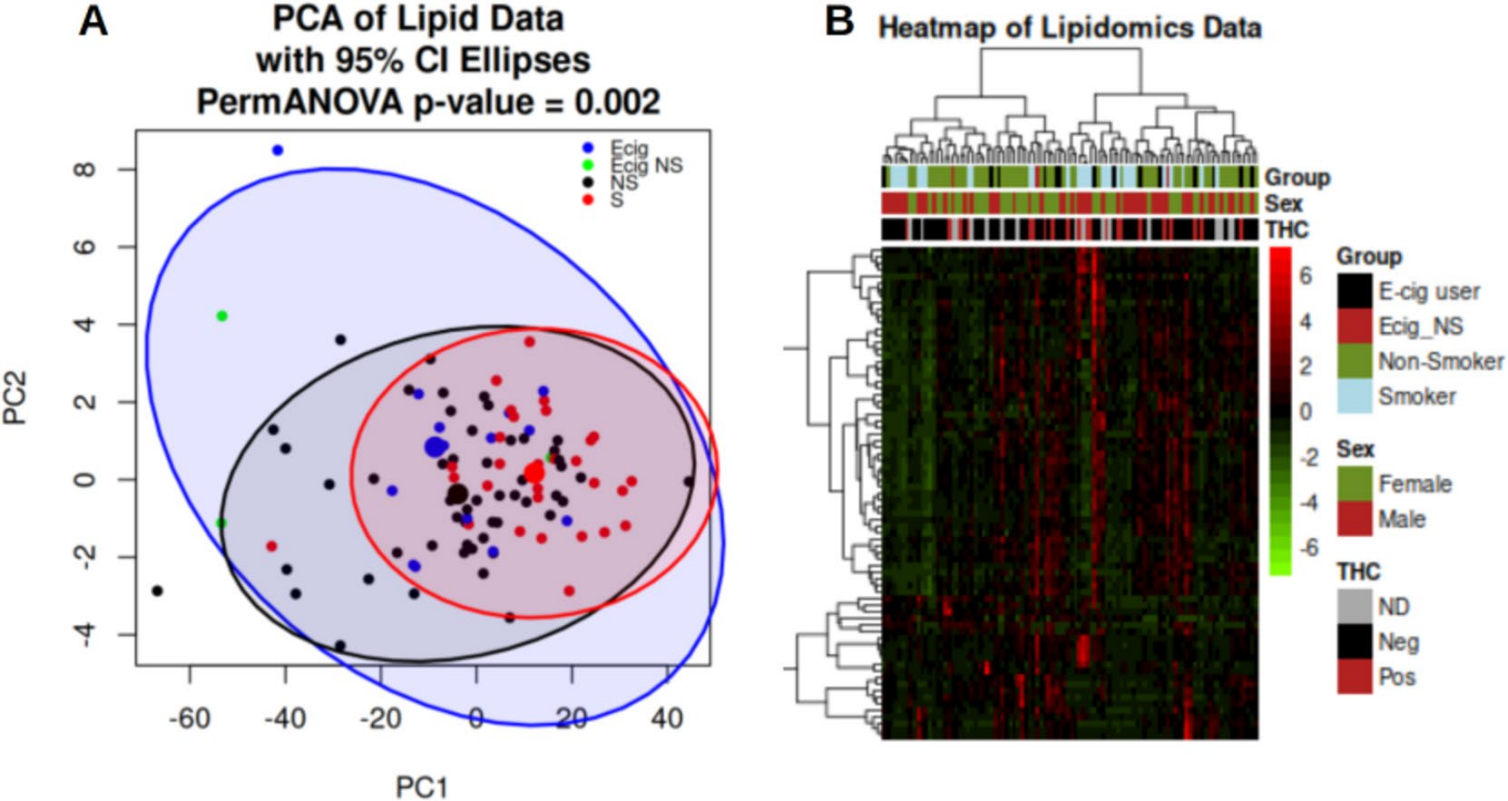
PCA and Heatmap of Lipid Data (all lipids). **A** Principal Component Analysis (PCA) was performed using Euclidean distance on lipidomic data to reduce dimensionality and visualize major sources of variance among samples. Ellipses are the 95% confidence intervals for Non-Smokers (NS), Smokers, and E-cig = E-cigarette users (3 NS Ecig included). PermANOVA was employed to test for differences between the groups. **B** Heatmap employed Ward clustering on correlations, with scaling by row and both columns and rows clustered

Data were available for 75 lipids. Figure S1 displays the histogram of a feature displaying the right skewed and LOD data, as well as the distribution of LOD values across features. Log transformation of the data results in an approximately normal distribution, with the exception of the LOD values. Based on similar distributions across the data, the left-censored log normal model was chosen for analyses of the lipidomics data. Figure 2 displays the unsupervised principal components analysis (PCA) and heat map (Ward clustering on correlations) using all lipid features. It can be seen from the PCA that, in general, a gradient exists on PC1 from left to right of non-smokers to smokers, with ECIG users approximately in the middle (PERMANOVA p-value < 0.01). The heat map shows no obvious clustering by group.

### Cross-sectional analyses

#### Lipid species and Smoking/Vaping status

**Fig. 3:**
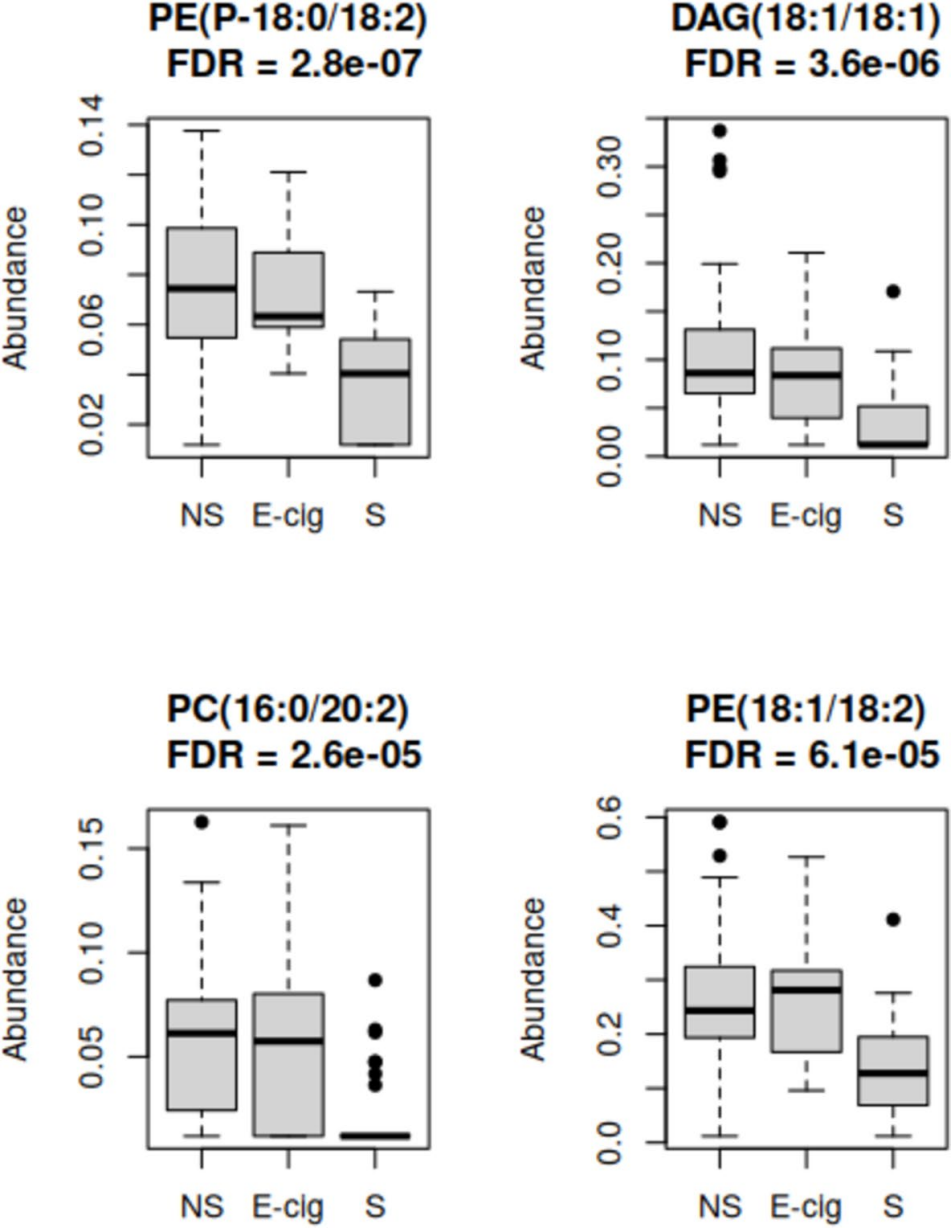
Boxplots of the top four lipids statistically significantly associated with smoking group in the cross sectional study. FDR q-value is for ANOVA of the left censored log-normal model. NS = Non-Smokers, E-cig = E-cigarette users, S = Smokers

**Fig. 4:**
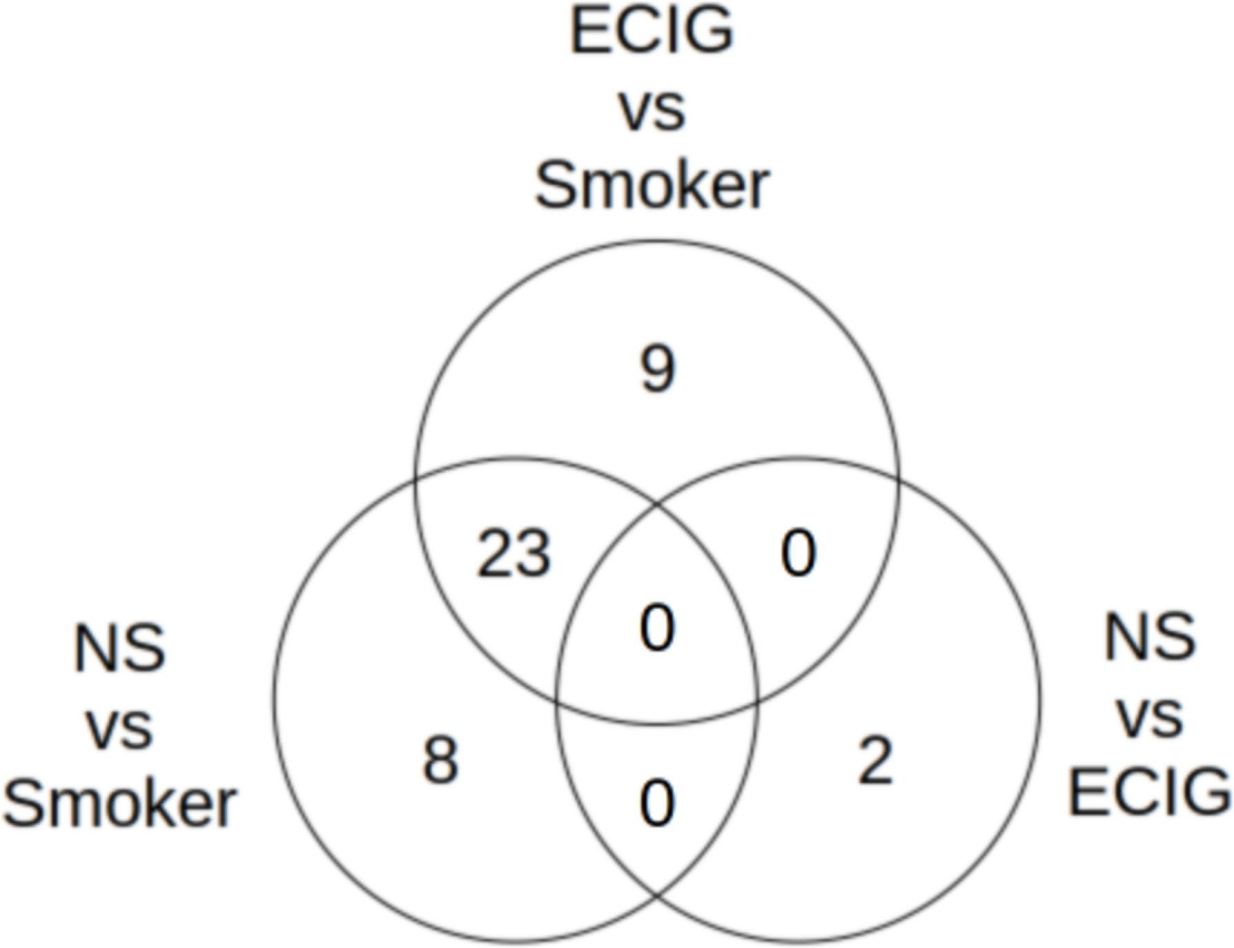
Venn Diagram of pairwise comparisons of omnibus significant features using the left-censored log-normal model. Significance threshold for these tests is p = 0.017. ECIG=E-cigarette; NS = Non-Smokers

Left censored log-normal models were fit with lipid feature as the dependent variable and smoking status as the independent variable. Considering FDR = 0.1, 43/75 lipid features were significantly associated with smoking status (top hits in Table 2, Fig. 3; full results in Table S1). In the pairwise follow-up analyses, 31 of these were significant (p < 0.017) comparing non-smokers to smokers, 32 significant comparing ECIG users vs. smokers, and two were significant comparing non-smokers to ECIG users, (PC(14:0/18:1) (higher in ECIG users) and PC(18:0/14:0) (higher in ECIG users) (Table S1). The Venn diagram in Fig. 4 suggests that differences from smokers appear to drive the across-group results, in most cases. The features unique to the pairwise comparisons were for non-smokers vs. smokers: CE (lower in smokers), CER(16:1) (higher in smokers), DAG(16:0/18:2) (lower in smokers), DAG(18:0/18:1) (lower in smokers), PE(P-16:0/18:2) (lower in smokers), PE(P-16:0/22:4) (higher in smokers), PE(P-18:0/18:1) (lower in smokers), and SM(16:0) (higher in smokers); for ECIG users vs. smokers: PC(16:0/16:1) (lower in smokers), PC(16:0/18:1) (lower in smokers), PC(18:0/18:1) (lower in smokers), PC(18:0/18:2) (lower in smokers), PC(18:1/16:1) (lower in smokers), PC(18:1/18:1) (lower in smokers), PC(18:1/18:2) (lower in smokers), PC(18:1/20:4) (lower in smokers), and PE(O-18:0/20:4) (lower in smokers); and for ECIG users vs. non-smokers: PC(14:0/18:1) (higer in ECIG users), and PC(18:0/14:0) (higher in ECIG users). For the multivariable models for the significant features, which include sex, THC, and age as covariables, 81 subjects had complete data and therefore could be included. For these models, 39/43 features were significant for the omnibus test (FDR = 0.1; Table S2).

**Table 2:** Top lipids associated with smoking group (ANOVA). ECIG = E-cigarette

**Feature**	**ECIG vs Non-Smoker estimate**	**Smoker vs Non-Smoker estimate**	**ANOVA raw P-value**	**FDR**
PE(P-18:0/18:2)	−0.029	−0.974	3.67E-9	2.75E-07
DAG(18:1/18:1)	−0.196	−1.609	9.53E-8	3.57E-06
PC(16:0/20:2)	−0.06	−1.713	1.04E-6	2.60E-05
PE(18:1/18:2)	0.073	−0.833	3.23E-6	6.06E-05
PE(18:1/18:1)	0.108	−0.729	2.98E-5	4.47E-04
SM(16:0)	0.235	0.729	7.48E-5	9.35E-04
TAG	0.036	−0.52	1.22E-4	1.21E-03
DAG(16:0/18:1)	−0.056	−1.066	1.29E-4	1.21E-03
LPC(16:0)	0.061	−0.794	2.23E-4	1.85E-03
PE(P-18:1/20:4)	0.192	−0.516	2.47E-4	1.85E-03

#### Lipid species and urinary biomarkers

Because smoking status was ascertained by self-report and may not reflect actual intake of inhaled toxicants, univariable models were also run with urine PG (a marker of ECIG use) or cotinine (a marker of nicotine intake for ECIG users and smokers) as the predictor of altered lipids. A total of 57 cross-sectional study subjects had PG data available. A clear outlier was present in the PG data and was excluded from analyses (Figure S2). Figure S3 A displays a boxplot of PG by smoking group, showing ECIG users had significantly higher PG levels than the other two groups (Kruskal-Wallis p = 0.000004). No lipid features were correlated with PG (FDR > 0.9). Full results are in Table S4. For cotinine analyses, 59 participants had data available (13 ECIG, 33 NS, 13 Smoker). As expected, smokers and ECIG users had higher levels of cotinine than the nonsmokers (Figure S3B) (Kruskal-Wallis p = 2.5 x 10-9). Additionally, cotinine significantly positively correlated with the following nine lipids (FDR > 0.1): CER(16:0), CER(24:1), CER, CER(24:0), PE(P-16:0/22:4), SM(22:0), PC(18:0/14:0), SM(16:0), and SM (Table S3).

#### Lipid species and inflammatory markers

Data were available for 14 lung biomarkers of inflammation (IM). Linear regression was employed with IM as the dependent variable, while lipid specie feature and smoking group where used as main effects, including main effect interactions. Of the 1050 models (14 IM x 75 lipid features), 179 had significant lipid main effects (FDR < 0.1). Sixty-five models had significant interaction effects (Table S4). Of the significant lipid effects, 42 were with IFNγ, 24 with IL8, 36 with IL10, 21 with IL6, 20 with IL12p70, 17 with IL4, and 19 with IL1β. No lipids were significantly associated (as a main effect) with macrophage %, neutrophil %, lymphocyte %, eosinophil %, TNFα, IL2, or IL13. The top 10 associations were with either IL1β or IL8, and included mostly CER or SM lipids. All of these top 10 also had significant lipid*smoking group interactions. The lipids significantly associated with the most inflammatory markers as main effects were SM(22:0) which associated with six inflammatory markers, and PC(18:0/18:1), PE(18:0/20:4), PE(P-16:0/18:2), SM, and SM(18:0) were associated with five inflammatory markers.

#### Lipid species and lipid-laden macrophages

A left-censored log-normal model was employed with lipid feature as the dependent variable and lipid-laden macrophages as the main effect. Batch effects in the LLM data was corrected using a linear model, with LLMs as the dependent variable, and using only the model residuals for the total of 61 subjects for further analyses. Ten lipid features negatively associated (FDR < 0.1) with the LLM residuals (Table S5). These included DAG, phosphatidylcholines (PC), phosphatidylethanolamines (PE), triacylglycerols (TAG), cholesteryl esters (CE), and SM. Because smoking status was highly associated with LLMs (p< 0.0001), lipid models were also generated including LLMs and smoking status as main effects, and their interactions. Independent of smoking status, there were no significant associations between the lipids and LLMs (FDR > 0.4), and no significant interaction effects (FDR > 0.9).

#### LC-MS/MS measured lipids and smoking/vaping status

LC-MS/MS lipidomics data for 97/98 subjects included CE, FC, TG, PC(32:0), PC, PE, PI, and SM. CE and TG were found in only two and one subjects, respectively, so analyses were not performed with these features. For the other six features, all 97 subjects had data which were approximately normal, so pairwise t-tests were performed to assess differences between the smoking groups, using significance threshold of FDR = 0.1 (3 pairwise*6 features=18 tests). Smokers had lower lipids levels in all significant tests (n=10), again indicating that smokers differ from the non-smokers and ECIG users in their lipid levels. SM was the only lipid not significantly different between smokers and ECIG users or non-smokers. Full results are in Table 3.

**Table 3:** LC-MS/MS measured lipids vs. smoking/vaping status (pairwise t-tests). ECIG = E-cigarette

**Dependent Variable**	**Group1**	**Group2**	**Group1 Mean**	**Group2 Mean**	**Raw P-value**	**FDR**
Phosphatidylinositol (PI) (nmol/mL)	Non-Smoker	Smoker	2.18	1.08	1.27E-07	2.29E-06
32:0-PC(nmol/mL)	Non-Smoker	Smoker	32.87	24.16	1.05E-05	9.45E-05
Phosphatidylcholine (PC) (nmol/mL)	Non-Smoker	Smoker	57.23	42.56	1.45E-04	8.70E-04
Phosphatidylcholine (PC) (nmol/mL)	ECIG	Smoker	61.04	42.56	2.52E-04	1.02E-03
Phosphatidylinositol (PI) (nmol/mL)	EIG	Smoker	2.02	1.08	3.04E-04	1.02E-03
Free Cholesterol (nmol/mL)	Non-Smoker	Smoker	11.60	7.83	3.40E-04	1.02E-03
32:0-PC (nmol/mL)	ECIG	Smoker	32.87	24.16	6.02E-04	1.55E-03
Phosphatidylethanolamine (PE) (nmol/mL)	ECIG	Smoker	6.06	4.18	1.20E-03	2.70E-03
Phosphatidylethanolamine (PE) (nmol/mL)	Non-Smoker	Smoker	5.59	4.18	1.47E-03	2.94E-03
Free Cholesterol (nmol/mL)	ECIG	Smoker	11.24	7.83	1.18E-02	2.12E-02
Sphingomyelin (SM) (nmol/mL)	Non-Smoker	Smoker	1.24	1.53	7.65E-02	1.25E-01
Sphingomyelin (SM) (nmol/mL)	ECIG	Non-Smoker	1.50	1.24	1.77E-01	2.65E-01
Phosphatidylethanolamine (PE) (nmol/mL)	ECIG	Non-Smoker	6.06	5.59	3.57E-01	4.94E-01
Phosphatidylcholine (PC) (nmol/mL)	ECIG	Non-Smoker	61.04	57.23	3.90E-01	5.01E-01
Phosphatidylinositol (PI) (nmol/mL)	ECIG	Non-Smoker	2.02	2.18	5.06E-01	6.07E-01
Free Cholesterol (nmol/mL)	ECIG	Non-Smoker	11.24	11.60	7.69E-01	8.65E-01
Sphingomyelin (SM) (nmol/mL)	ECIG	Smoker	1.50	1.53	8.97E-01	9.50E-01
32:0-PC (nmol/mL)	ECIG	Non-Smoker	32.87	32.87	1.00E+00	1.00E+00

#### Saturated/Unsaturated ratios and smoking/vaping status

Ratios for saturated/unsaturated values were computed for lipid classes with at least one of each type in the dataset. Because not all lipid classes in the dataset had both a saturated and unsaturated feature measure, these analyses were only performed for CER, SM, DAG, PC, and LPC. A linear model was used with log_10_(ratio) as the dependent variable and smoking group as the independent variable. CER, SM, and DAG were significantly associated with smoking group (FDR < 0.1; Table 4). Pairwise analyses reveals significantly higher (p < 0.017) saturated lipids in smokers versus ECIG and nonsmokers (Table 5). Importantly, none of the ratios were significantly different between ECIG users and non-smokers.

**Table 4:** Log10(Saturated/Unsaturated ratios) vs. smoking/vaping status (ANOVA). ECIG = E-cigarette

**Lipid Saturation Ratio**	**ECIG vs Non-Smoker Estimate**	**Smokers vs Non-Smoker Estimate**	**ANOVA raw P-value**	**FDR**
CER	0.031	0.172	3.46E-06	1.73E-05
SM	−0.009	0.118	1.90E-05	4.75E-05
DAG	−0.048	0.273	4.76E-05	7.93E-05
PC	−0.007	0.03	4.91E-01	5.77E-01
LPC	−0.035	0.049	5.77E-01	5.77E-01

**Table 5:** Log10(Saturated/Unsaturated ratios) vs. smoking/ECIG (E-cigarette) use status (followup pairwise t-tests)

**Lipid Saturation Ratio**	**Mean Non-Smoker**	**Mean ECIG**	**Mean Smoker**	**ECIG vs Non-Smoker raw P-value**	**Smoker vs Non-Smoker raw P-value**	**ECIG vs Smoker raw P-value**
CER	0.11	0.14	0.28	4.23E-01	7.36E-07	1.41E-03
DAG	−0.15	−0.20	0.12	5.31E-01	4.70E-05	2.45E-04
SM	0.29	0.28	0.40	7.59E-01	1.21E-05	2.69E-04

#### Ontological analyses

**Fig. 5:**
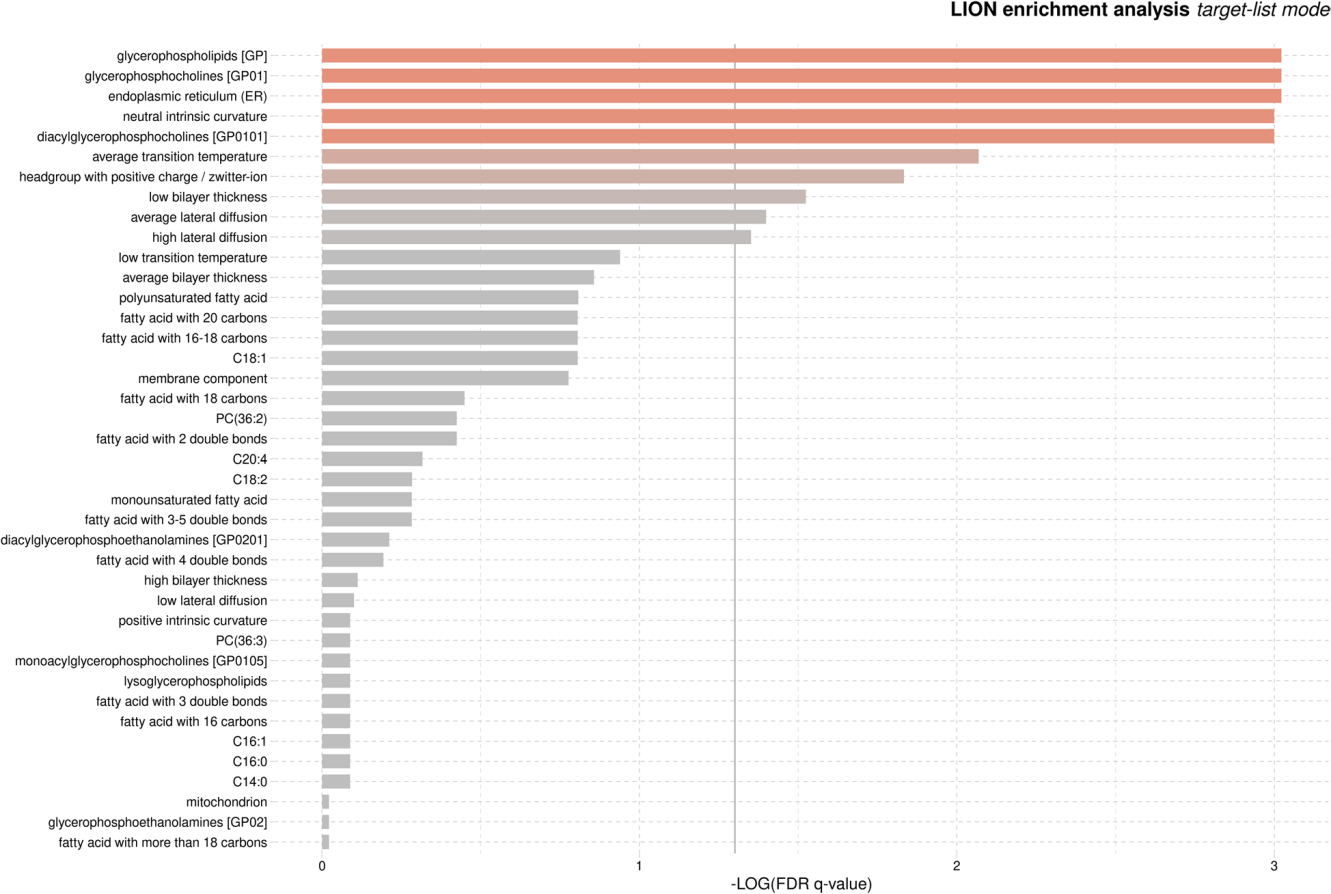
Plot of the significance of ontological categories from the LION enrichment analysis for ECIG (E-cigarette) users vs. smokers in the cross sectional study (generated by http://www.lipidontology.com/)

Using lipid specie features, the web-based LION analysis (Target List Mode) was employed to identify over-represented lipid ontological categories pairwise by smoking group (pairwise full lipid results in Table S6). The 75 lipids tested were used as the background lipid pool for determining over-representation, and lipids pairwise significant at FDR <= 0.05 were used to identify significant features. For the ECIG vs. non-smokers, none of the lipids were significant at this level so the LION analyses were not performed. For non-smokers vs. smokers, none of the ontological categories were significant (FDR > 0.6). For the comparison of ECIG users vs. smokers, ten ontological categories were significant (FDR < 0.05; Fig. 5). These included (from most to least significant) glycerophospholipids, endoplasmic reticulum, glycerophosphocholines, diacylglycerophosphocholines, neutral intrinsic curvature, average transition temperature, headgroup with positive charge/zwitterion, low bilayer thickness, average lateral diffusion, and high lateral diffusion.

### Clinical trial

For the clinical trial, non-smokers underwent serial bronchoscopy, n=30 at baseline and then 15 were randomized to the nicotine- and flavor-free ECIG use and 15 were randomized as no-use controls. All underwent a second bronchoscopy after 4 weeks of ECIG use or no use. As in the cross-sectional analyses, a left-censored log-normal model was employed. For these models, follow-up lipid measurement was used as the dependent variable, baseline lipid measure was included as a covariable, and intervention group was the main effect. ECIG use was not associated with any changes in lipid features (FDR > 0.8; Table S7). PG data were available for all 30 clinical trial subjects at both baseline and follow-up. As with the cross-sectional analyses, the same subject was removed from the clinical trial analyses because of being an outlier in the distribution of PG (Figure S2). None of the lipids were significantly associated (FDR > 0.9; Table S8) with PG levels across intervention groups nor within ECIG users only. For the inflammatory markers, none of the lipid main effects nor interaction effects for any lipid/IM combinations were significant (FDR > 0.4; Table S9) across groups (group and group*lipid interaction in the model). None of the lipid features were significantly associated with changes in LLMs (FDR > 0.4; Table S10). None of the LC-MS/MS lipids were significantly associated with ECIG use (FDR > 0.4; Table S11), and none of the saturated/unsaturated comparisons were significant (FDR > 0.7; Table S12).

## Discussion

This is the first study to assess the effects of ECIG use and smoking on the lung lipidome of healthy individuals, enhanced by using complementary cross-sectional and clinical trial designs. We identified differences in lipid species in the cross-sectional study, including two lipids that differed between ECIG users and non-smokers. We also found many lipids that were different between smokers vs. ECIG users and non-smokers, which provides some evidence that cigarette smoking potentially leads to changes in lung lipid profiles. Associations between lipid species and inflammatory markers were also identified, including lipid*group interactions, indicating different lung inflammatory states by smoking status (ECIG vs. smoker vs. non-smoker). Additionally, smokers had a higher saturation ratio in CER, DAG, and SM than non-smokers and ECIG users. Taken together, these findings indicate that smoking may have a larger effect on lipid levels in the lung than ECIG use, which may have a much smaller, and in some cases different effect. This is similar to our previous findings with different biomarkers [6, 9, 43], which found that molecular differences associated with smoking are typically large compared to ECIG users and non-smokers, whereas differences between ECIG users and non-smokers are more modest where they exist. In our clinical trial of ECIG use among non-smokers, significant differences between groups were not identified, likely because of the short treatment duration (4 weeks), smaller numbers of puffs per day compared to the number of daily puffs for exclusive ECIG users (40 from the trial vs 160 from the cross-sectional study), and the more modest effects seen between ECIG users and non-smokers.

### Cross-sectional study

#### Primary comparison

Of lipids that differed by smoking status with the omnibus test, most were significant or suggestive of associations in the pairwise tests between smokers versus the other two groups, indicating potential global lipid disruption in the lung from smoking cigarettes. In ECIG users compared to non-smokers, we identified two significantly different lipids, PC(14:0/18:1) and PC(18:0/14:0), which may be unique ECIG-related lipids and differ from smoking associations.

For the omnibus significant lipids, lipid levels in smokers were lower than that of non-smokers and ECIG users. This is consistent with prior findings showing lower levels of lung lipids, including surfactant lipids in COPD [15]. Specifically, sphingolipids, a primary component of pulmonary surfactant [44], were lower present in COPD and former smokers. Despite an older study population of their study (vs young adults in the current study), consistent reductions were also replicated in a smoke-exposed mouse model, indicating this observation may not be age-specific. Further, their study additionally found that lower lipid levels correlated with lower lung function, a hallmark of COPD, implicating a potential clinical contribution of lower lung lipids to pulmonary diseases.

Three PCs appeared to be elevated in ECIG use, namely PC(14:0/18:1), PC(18:0/14:0), and PC(18:1/20:4) that were significantly or marginally associated (p < 0.05) with both ECIG users vs. smokers and ECIG users vs. non-smokers, but not for smokers vs. non-smokers. Other PCs, in general, were higher in ECIG users than in non-smokers. PC is a major phospholipid that makes up to 80% of surfactant lipids [45], which are involved in reducing surface tension in the lungs [15]. ECIGs have previously been shown to increase PCs, as well as other phospholipids, in BAL cells from ECIG-exposed mice, and to upregulate the expression of PC-synthesizing enzyme genes [46]. Additionally, PC metabolism has been implicated in cancer/immune cell cross-talk in the tumor micro-environment [47]. For example, LysoPC can modulate immune cell recruitment and activation, be involved in inflammatory signaling [48], and inhibit lung cancer proliferation within the tumor micro-environment [49]. This supports further research on potential ECIG-related health effects, specifically in immune responses and cancer.

#### Inflammatory markers

Much evidence exists that lipids affect inflammation [50, 51]. In the current study, many lipids (~17% of the lipid/inflammatory marker combinations) were significantly associated with inflammatory markers after adjusting for smoking group, along with significant interactions within smoking groups, indicating that the relationship between the lipids and inflammation differs by smoking group. The top associations were found primarily with IL-1β and IL-8. Studies [52, 53], including ours [6], showed IL-1β levels were higher in smokers’ BAL compared to non-smokers’ BAL [54]. Moreover, we reported significantly higher levels of IL-1β in ECIG users compared to non-users (59% higher), using a subset of study participants from this study [6]. IL-1β is a pro-inflammatory cytokine that plays a key role in initiating the inflammatory response in pulmonary diseases [55]. Given the critical role of IL-1β in tumor growth and metastasis in lung cancer [56], IL-1β inhibitor is being evaluated as an anti-inflammatory therapy in lung cancer patients [57]. IL-8 is also a pro-inflammatory cytokine that plays a role in lung defense [58]. The subset of samples from this study showed a significantly higher level of IL-8 in the BAL of smokers compared to non-smokers. Separately, higher amounts of IL-8 are found in the BAL, sputum, and lung tissue of those with chronic lung diseases [58]. Given that our study participants are young adults with no history of pulmonary diseases, our cross-sectional findings highlight the need to investigate how smoking- and potentially ECIG-altered lipids may drive lung inflammation and predispose to pulmonary diseases.

#### Lipid-Laden macrophages

The ECIG or Vaping Product Use-Associated Lung Injury (EVALI) outbreak was discovered to be caused by adulterated cannabis vaping containing vitamin E acetate [59]. LLMs were initially thought to be involved in the pathology of EVALI but were later identified in both smokers and ECIG users [43, 53]. Importantly, our group reported a unique link between ECIG use and LLM, suggesting a novel lipid-driven pathway of ECIG-related pulmonary health [60]. Moreover, an ECIG-exposed mouse model showed lipid accumulation in alveoli [46], supporting further systematic assessment of the airway’s lipidome in the lungs of ECIG users. Therefore, we investigated whether lipids were associated with LLMs in BAL of smokers, ECIG users, and non-smokers. While ten lipid features were associated with LLMs, none of the lipids were significant in within-group analyses, indicating a smoking effect. Given the cross-sectional study design, further studies are warranted to define a potential relationship between smoking-related lipids and accumulating alveolar macrophages.

#### Saturated/Unsaturated

For the analysis of saturation of lipids, significant differences were only observed in comparisons with the smoking group. For each of the significant lipids (CER, DAG, and SM), saturation was significantly higher in the smoking group, with non-smokers and ECIG users being similar. This is in contrast to a previous study [46] of mice exposed to ECIGs, where an increase in lipid saturation was observed in the ECIG-exposed mice.

### Clinical trial

In the clinical trial of ECIG use among non-smokers, no significant changes with ECIG use were identified, even when assessing in the context of ECIG use (measured by urinary exposure biomarkers, including PG or cotinine). Thus, the clinical trial was not able to replicate the cross-sectional analyses. Given that most of the significant associations in the cross-sectional study were results of differences from the smoker's group, this does not contradict the cross-sectional results. Additionally, this ECIG use trial was only performed for four weeks of ECIG use with much smaller numbers of puffs per day compared to the number of daily puffs for exclusive ECIG users (40 from the trial vs 160 from the cross-sectional study). Thus, exposure was likely not long enough to observe long-term exposure effects that might be identified in the cross-sectional study.

Although some effects specific to ECIG use were noted in the cross-sectional analyses, overall, ECIG users appear to be more similar to non-smokers than to smokers when considering the lipidomics data as a whole, as we have seen previously with other biomarkers [6]. However, two lipids, (PC(14:0/18:1) and PC(18:0/14:0), were significantly different between ECIG users and non-smokers in pairwise follow-up tests. These PCs have been shown to have a similar gel-to-liquid melting temperature as PC(16:0/16:0), the major constituent of lung surfactant [61]. However, PC(14:0/18:1) and PC(18:0/14:0) are asymmetrical in the length of the esterified FAs, which may promote protein anchoring on cell walls, extracellular vesicles, and lipid droplets [62]. Whether these properties of PC(14:0/18:1) and PC(18:0/14:0) are relevant to the physiology and pathology of the lung in ECIG use requires further study.

One of the main strengths of our study is that the samples were obtained from the target organ of smoking and ECIG exposure and lung diseases. Additionally, we employed a censored log-normal model to account for LOD values, rather than using imputation. Both cross-sectional and clinical trial data (clinical trial for ECIG) were included in the study. However, there are also some important limitations to our study. All of the significant associations identified were in the cross-sectional study. Causal inferences cannot be concluded from cross-sectional studies. In the current study, multivariable models were conducted using gender, THC positivity, and age as covariables. It is possible that other, currently unknown or unrecorded covariables could alter or mediate the associations herein. Numbers for the current study were modest (especially the ECIG group). Larger studies are needed to validate these results and to obtain more precise estimates.

## Conclusions

In summary, this novel study of target organ (lung) exposure found smoking to be associated with altered lipid levels as compared to both non-smokers and ECIG users, and ECIG-specific associations were also identified. As lipids have been identified as having potential roles in inflammation and cancer, the results herein in the lungs are important for identifying additional potential means through which cigarette smoking and ECIG use alter the lung. Furthermore, because ECIG use may serve as a risk reduction strategy for some smokers, the identified lipidome differences between smokers, ECIG users, and non-smokers suggest the need for future studies characterizing the role of smoking and ECIG-related lipid alteration in pulmonary disease.

### Disclaimer

The findings and conclusions in this manuscript are those of the authors and do not necessarily represent the official position of the Centers for Disease Control and Prevention (CDC). Use of trade names is for identification only and does not imply endorsement by the CDC, the Public Health Service, or the U.S. Department of Health and Human Services.

## Supplementary Information

Supplementary material 1.

Supplementary material 2.

Supplementary material 3.

Supplementary material 4.

Supplementary material 5.

Supplementary material 6.

Supplementary material 7.

Supplementary material 8.

Supplementary material 9.

Supplementary material 10.

Supplementary material 11.

Supplementary material 12.

Supplementary material 13.

Supplementary material 14.

Supplementary material 15.

Supplementary material 16.

## Data Availability

The datasets used and/or analysed during the current study are available from the corresponding author on reasonable request.

## Abbreviations

ECIG: electronic cigarette
PG: propylene glycol
VG: vegetable glycerin
LLM: lipid-laden macrophages
BAL: bronchoalveolar lavage
LOD: limit of detection
