# Supplementary figures and images for: Lung lipids associated with smoking and ECIG use in a cross-sectional study and clinical trial
Source: Respir Res. 2025 May 20;26:193. doi: 10.1186/s12931-025-03267-w (PMC12093903; doi:10.1186/s12931-025-03267-w)

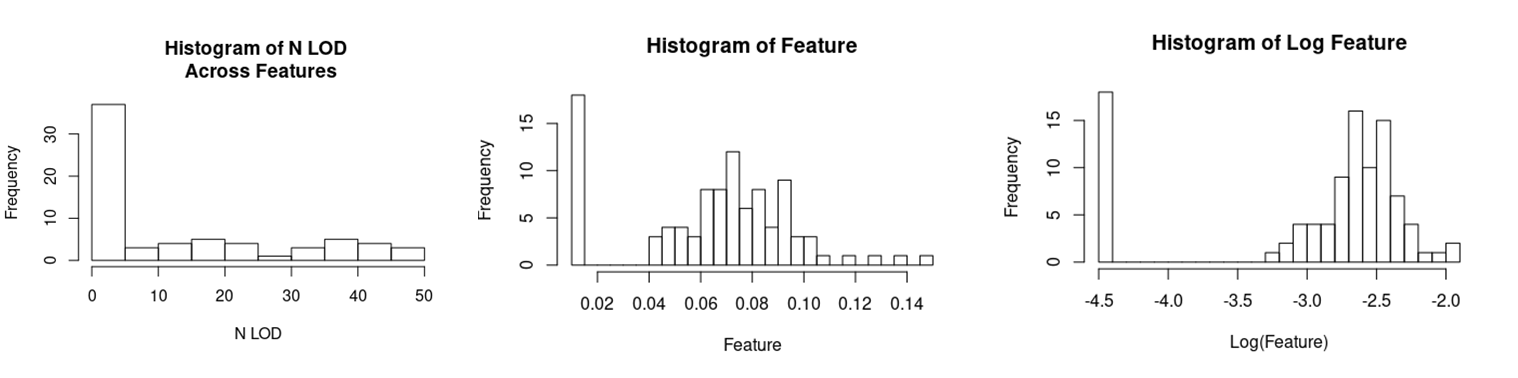

Supplement: Supplementary file 2 — Supplementary material 2. [file 12931_2025_3267_MOESM2_ESM.tif]

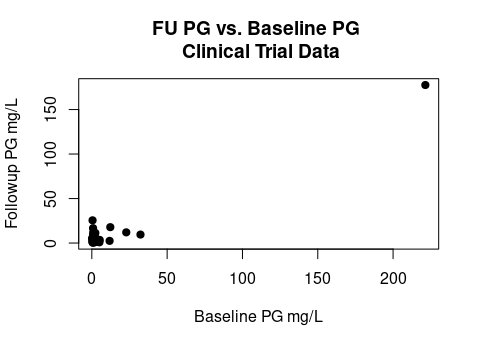

Supplement: Supplementary file 3 — Supplementary material 3. [file 12931_2025_3267_MOESM3_ESM.tif]

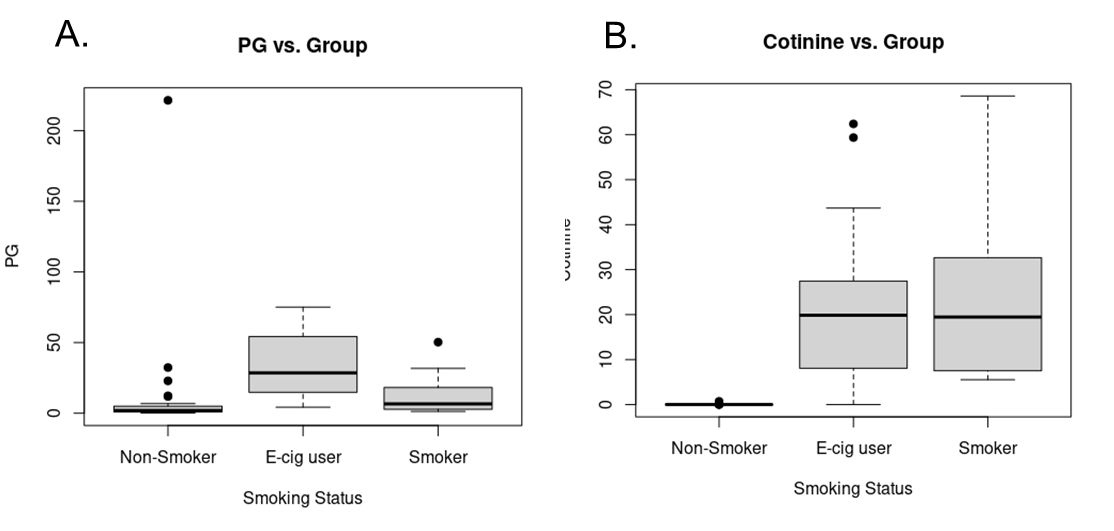

Supplement: Supplementary file 4 — Supplementary material 4. [file 12931_2025_3267_MOESM4_ESM.tif]
